# Regional anticoagulation with heparin of an extracorporeal CO_2_ removal circuit: a case report

**DOI:** 10.1186/s13256-019-2051-6

**Published:** 2019-05-03

**Authors:** Jacopo Tramarin, Andrea Cortegiani, Cesare Gregoretti, Filippo Vitale, Cesira Palmeri, Pasquale Iozzo, Francesco Forfori, Antonino Giarratano

**Affiliations:** 1 Department of Surgical, Oncological and Oral Science (Di.Chir.On.S.), Section of Anesthesia, Analgesia, Intensive Care and Emergency, Policlinico Paolo Giaccone, University of Palermo, Palermo, Italy; 2 Department of Anesthesia and Intensive Care, University of Palermo, Palermo, Italy; 3 Department of Anesthesia and Intensive Care, University of Pisa, Pisa, Italy

**Keywords:** Extracorporeal CO_2_ removal, Heparin regional anticoagulation, Continuous venovenous filtration

## Abstract

**Background:**

Extracorporeal carbon dioxide removal is an increasingly used respiratory support technique. As is true of all extracorporeal techniques, extracorporeal carbon dioxide removal needs proper anticoagulation. We report a case of a patient at risk of bleeding complications who was treated with extracorporeal carbon dioxide removal and anticoagulated with a regional technique.

**Case presentation:**

A 56-year-old Caucasian man with a history of chronic obstructive pulmonary disease exacerbation required extracorporeal carbon dioxide removal for severe hypercapnia and acidosis despite mechanical ventilation. The extracorporeal circuit was anticoagulated using a regional heparin technique to limit the patient’s risk of bleeding due to a low platelet count. The patient underwent 96 h of effective extracorporeal carbon dioxide removal without any adverse events. He was successfully weaned from extracorporeal carbon dioxide removal. During the treatment, no bleeding complications or unexpected circuit clotting was observed.

**Conclusions:**

The use of regional heparin anticoagulation technique seems to be feasible and safe during extracorporeal carbon dioxide removal.

## Background

Extracorporeal carbon dioxide (CO_2_) removal (ECCO_2_r) is a technique that allows artificial respiratory support by providing CO_2_ clearance through an extracorporeal circuit, thus unloading the respiratory system. ECCO_2_r has been used during acute respiratory distress syndrome (ARDS) to allow protective ventilation without increasing partial pressure of carbon dioxide (PaCO_2_) [1]. Other indications include chronic obstructive pulmonary disease (COPD) exacerbation and refractory respiratory acidosis [1]. Because blood has to proceed through an extracorporeal circuit and filters, proper anticoagulation is needed.

The best anticoagulation strategy for ECCO_2_r is still debated [2, 3]. We present a case of a patient with COPD with acute respiratory failure who was treated in an intensive care unit (ICU) with ECCO_2_r support using a regional heparin anticoagulation method.

## Case presentation

Our patient was a 56-year-old Caucasian married man with height 172 cm, weight 75 kg, and body mass index 25.4 kg/m^2^. He was admitted to our emergency department for severe dyspnea and desaturation.

The patient had a history of heavy smoking (30 pack-years) and no alcohol intake. In the last year, he had had two hospitalizations for acute exacerbation of COPD and was classified as Global Initiative for Chronic Obstructive Lung Disease class C. He was admitted to the ICU and eventually was tracheostomized. After his ICU stay, he was decannulated and actually showed a former closed tracheal stoma. Moreover, he had type 2 diabetes mellitus and hypertension, and he presented with a former closed tracheal stoma after his last ICU admission for COPD exacerbation. His medication history included ramipril, pantoprazole, and inhalation indacatarol/glycopyrronium.

**Fig. 1:**
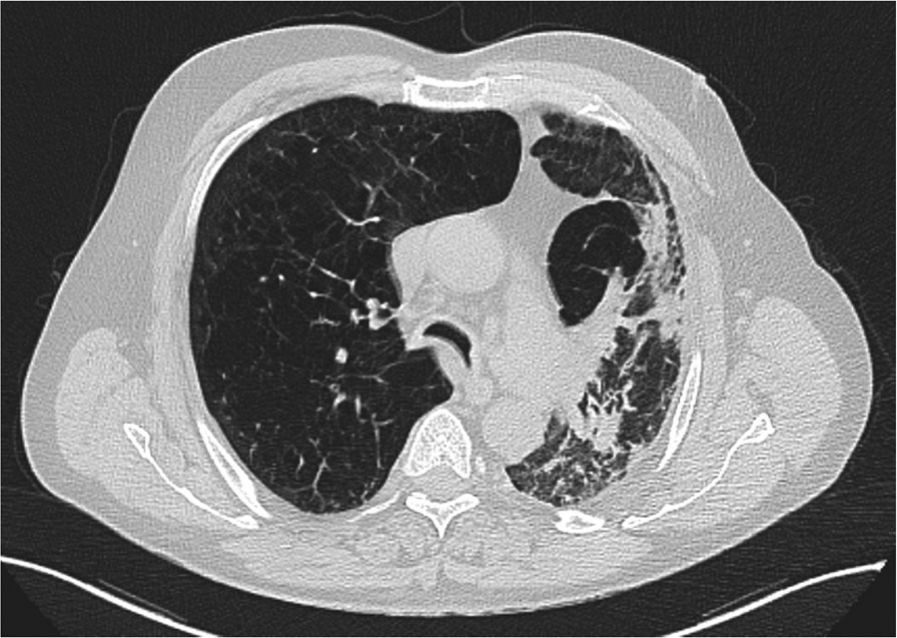
Chest computed tomography performed on day 1 showing diffused centrilobular emphysema and a fibrothorax aspect. Air bronchograms were evidenced bilaterally at bases

At arrival, the patient showed hypercapnic respiratory acidosis (pH 7.24, partial pressure of oxygen 45 mmHg, PaCO_2_ 70 mmHg, HCO_3_^−^ 32 mEq/L). Standard medical therapy and noninvasive ventilation (NIV) were immediately started. The result of his neurological examination was normal with a Glasgow Coma Scale score of 15. His heart rate was 106 beats/min, peripheral oxygen saturation was 86%, noninvasive blood pressure was 135/85 mmHg, and body temperature was 37.8 °C. A chest computed tomographic scan showed a centrilobular emphysema and a bilateral fibrothorax (Fig. 1).

A few hours after admission, the patient was intubated for worsening mental status and worsening respiratory acidosis (pH 7.18, PaCO_2_ 85 mmHg). Mechanical ventilation in pressure support mode was started in the ICU associated with salmeterol and fluticasone 50 μg/100 μg inhalational therapy every 8 h. Sedation was obtained by titrating propofol infusion to obtain a Richmond Agitation-Sedation Scale score of − 1. On the basis of white blood cell count of 22 × 10^9^/L, high procalcitonin serum level 12 ng/ml, and strong suspicion of a pulmonary infection, bronchoalveolar lavage was collected, and intravenous broad-spectrum empiric antibiotic therapy with piperacillin-tazobactam 4.5 g every 8 h and vancomycin 500 mg every 6 h was started. After 72 h, qualitative bronchial cultures showed a negative Gram stain and heavy growth of *Pseudomonas aeruginosa*. At this point, intravenous cefepime 2 g every 8 h was started.

On day 4 after admission, owing to the severity of lung infection, hypercapnic respiratory acidosis worsened to pH 6.98 and PaCO_2_ 157 mmHg despite profound sedation and the maximization of minute alveolar ventilation. A low platelet count of 50,000 cells/μl was recorded. ECCO_2_r was started through a 16-French dialysis bilumen catheter inserted into the right femoral vein using continuous renal replacement therapy (CRRT) (Diapact® system; B. Braun Medical, Milan, Italy) with a Diacap Acute® filter (B. Braun Medical). The extracorporeal circuit was regionally anticoagulated with heparin administered prefilter and protamine sulfate administered postfilter (Fig. 1).

Table 1 shows blood gas analyses before, during, and after ECCO_2_r treatment. The patient’s PaCO_2_ dropped to 54 mmHg and pH increased to 7.21 after 6 h of treatment. The Diacap Acute® filter was replaced every 24 h during ECCO_2_r. On day 6 after admission, because PaCO_2_ consistently less than 60 mmHg, ECCO_2_r weaning was attempted, and the patient was switched again into pressure support mode. ECCO_2_r was started again 2 h later because of a rise in PaCO_2_. On day 7 after admission, the patient was successfully weaned from ECCO_2_r. Under pressure support ventilation, he was able to maintain an acceptable PaCO_2_ level (59 mmHg). On day 9 after admission, the patient became febrile with associated hypotension and increased serum lactate level. Multiple organ failure developed during the following 48 h. On day 11 after admission, blood cultures evidenced multidrug-resistant *P. aeruginosa*.

**Table 1:** Blood gas analyses before, during, and after extracorporeal carbon dioxide removal with regional anticoagulation

	Blood gas analyses										
	ED	ICU									
	Day 1	Day 1	Day 2	Day 3	Day 4	Day 5	Day 6	Day 7	Day 8	Day 9	Day 10
pH	7.24	7.18	7.19	7.12	6.99	7.28	7.30	7.31	7.30	7.28	7.22
PaO_2_	70	90	84	125	101	99	140	119	111	132	143
PaCO_2_	129	114	142	123	157	61	56	55	58	56	55
					Post ECCO_2_r	Filter change	Weaning trial	Weaning trial			
				pH	7.29	7.31	7.24	7.30			
				PaO_2_	145	108	129	113			
				PaCO_2_	54	52	73	59			

ECCO_2_r was started at day 4

*Abbreviations: ECCO*_*2*_*r* extracorporeal carbon dioxide removal, *ED* Emergency department, *ICU* Intensive care unit, *PaCO*_*2*_ Partial pressure of carbon dioxide, *PaO*_*2*_ Partial pressure of oxygen

### Extracorporeal circuit and anticoagulation technique

**Fig. 2:**
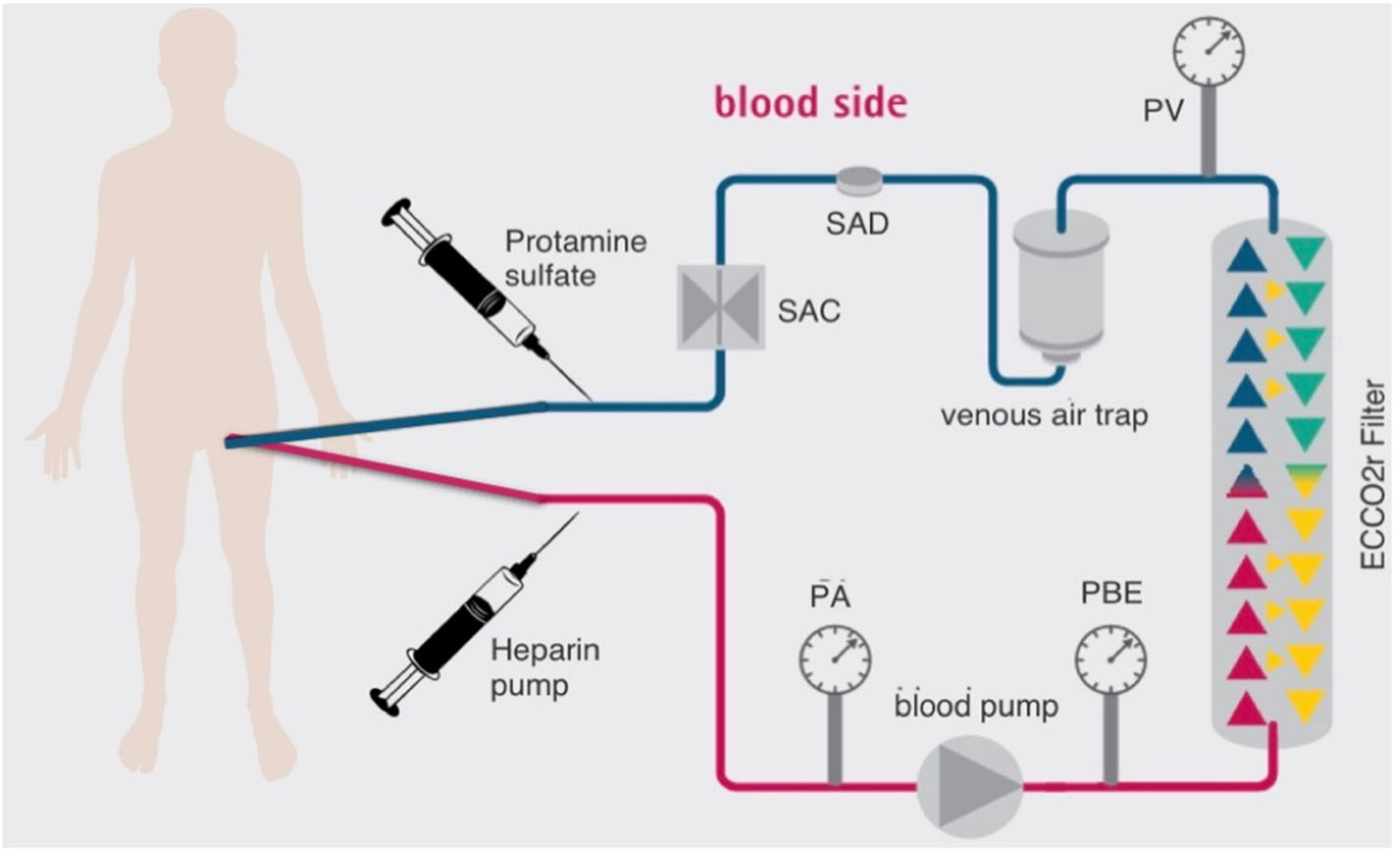
Schematic representation of extracorporeal carbon dioxide removal with regional heparin protamine anticoagulation. *PA* Arterial pressure, *PBE* Prefilter pressure, *PV* Venous pressure, *SAD* Safety air detector, *SAC* Safety clamp

The extracorporeal circuit was primed two times, first with 1 L of NaCl 0.9% + 10,000 IU of unfractionated heparin and then with 1 L of NaCl 0.9%. A 500-IU/ml heparin solution was prepared and infused prefilter at 0.15 IU/ml/h of blood flow (Fig. 2). A protamine concentration of 5 mg/ml was infused to match the heparin infusion rate [4]. ECCO_2_r was started at a blood flow of 300 ml/min and increased slowly to 450 ml/min to maximize CO_2_ removal.

## Discussion

To the best of our knowledge, this case report is the first in the literature of regionally anticoagulated ECCO_2_r in a patient with COPD with acute respiratory failure. The main finding in this case report is the efficacy and safety of using ECCO_2_r with a heparin regional anticoagulation regimen.

Severe hypercapnia has several detrimental pathophysiological effects. Accumulation of CO_2_ causes acidosis, reduced consciousness, and an increase in cerebral blood flow due to vasodilation. Moreover, hypercapnic acidosis causes myocardial depression and pulmonary vasoconstriction, raising the risk of both right and left ventricular failure [5].

First conceived in the late 1970s, ECCO_2_r has been used as an adjuvant respiratory treatment for patients with severe ARDS ventilated with protective ventilation but in need of increasing CO_2_ elimination [6] and reducing tidal volumes to limit lung damage. More recently, ECCO_2_r has been used for the treatment of severe COPD exacerbations to avoid intubation [7] or in weaning from mechanical ventilation and as a supportive treatment of severe respiratory hypercapnic acidosis [8].

Our patient had an acute COPD exacerbation for which NIV treatment failed in the emergency department. Mechanical ventilation in the ICU was challenging because of the patient’s obstructive restrictive pattern. Minute ventilation was increased to 13 L/min, reaching safe pressure limits (driving pressure < 15 cm H_2_O). However, acceptable pH and CO_2_ levels were not obtained. Because standard medical treatment and mechanical ventilation were unsuccessful, ECCO_2_r was started to improve the life-threatening hypercapnic acidosis and potentially injurious mechanical ventilation.

As is true of most continuous venovenous blood purification systems, ECCO_2_r needs anticoagulation to ensure circuit and filter patency. The best anticoagulation strategy is still debated in the literature. A recent systematic review that summarized the evidence about anticoagulation during CRRT indicated that regional citrate-based anticoagulation was the most efficient and safe method [2]. However, the citrate-based anticoagulation regimen can induce hypocalcemia and metabolic alkalosis. Moreover, in order to reach efficacious venovenous ECCO_2_r, a minimum blood flow of 350 ml/min is necessary. Therefore, citrate could hardly be the anticoagulation mode of choice because of the excessive fluid volume and metabolic load for the body. Indeed, the amount of infused citrate could be above the hepatic metabolic limit, thus resulting in citrate intoxication. Last, postfilter calcium administration could require a massive dosage and a very high speed of infusion to correct the citrate-related hypocalcemia [9]. Potential hemorrhage was a major concern in our patient because of low platelet count. Thus, in our opinion, regional anticoagulation with heparin and protamine was the best regimen to reduce bleeding risk. Regional heparin-based anticoagulation is widely used in CRRT worldwide [4]. Nevertheless, in their randomized controlled trial conducted in 2015, Gattas *et al*. compared regional heparin with regional citrate for anticoagulation in CRRT and concluded that an equivalent safety profile and use of citrate allowed a longer CRRT filter life [10].

Regional heparin anticoagulation is not without risk. Because heparin is supposed to have a longer half-life than protamine, there is a risk of rebound bleeding in cases of incorrect titration. Furthermore, this technique exposes the patient to side effects caused by heparin, such as thrombocytopenia, and to the risks of protamine administration, such as anaphylaxis, hypotension, and pulmonary vasoconstriction [11]. Nevertheless, most of these drawbacks can be avoided with a slow protamine infusion and a precise heparin-neutralizing protocol.

This case report has limitations. First, we did not perform viscoelastic tests (rotational thromboelastometry or thromboelastography). Moreover, we did not evaluate the heparin-protamine complexes in the patient’s blood, so we do not know the serum half-life of the complexes. However, we did not detect any major or minor bleeding event. Daily platelet count and standard coagulation test results were stable and within normal range throughout the treatment.

## Conclusions

With the increasing use of ECCO_2_r, there is a need to increase its safety with regard to anticoagulation. Regional heparin anticoagulation may represent a feasible way to provide ECCO_2_r for CRRT. Adequately powered studies are needed to confirm these observations.

## Abbreviations

ARDS: Acute respiratory distress syndrome
CO_2_: Carbon dioxide
COPD: Chronic obstructive pulmonary disease
CRRT: Continuous renal replacement therapy
ECCO_2_r: Extracorporeal carbon dioxide removal
ICU: Intensive care unit
NIV: Noninvasive ventilation
PaCO_2_: Partial pressure of carbon dioxide
PaO_2_: Partial pressure of oxygen
